# NFE2L2 activator RS9 protects against corneal epithelial cell damage in dry eye models

**DOI:** 10.1371/journal.pone.0229421

**Published:** 2020-04-22

**Authors:** Yuka Matsuda, Mamiko Machida, Yasuhiro Nakagami, Takeshi Nakajima, Mitsuyoshi Azuma

**Affiliations:** 1 Senju Laboratory of Ocular Sciences, Senju Pharmaceutical Co., Ltd., Kobe, Japan; 2 Daiichi Sankyo Co., Ltd., Tokyo, Japan; University of Tasmania, AUSTRALIA

## Abstract

Oxidative stress may cause ocular surface damage during the development of dry eye. Mammalian cells have defense systems against oxidative stress. A central regulator of the stress response is nuclear factor-erythroid 2-related factor 2 (NFE2L2). NFE2L2 is activated by the novel triterpenoid RS9 (a biotransformation compound of RTA 402). The purpose of this study was to assess the efficacy of RS9 against dry eye using *in vitro* and *in vivo* models. Bioactivity was estimated by the induction of mRNAs for two NFE2L2-targeted genes: *NQO1* (prevents radical species) and *GCLC* (glutathione synthesis), using a corneal epithelial cell line (HCE-T). Protection against oxidation and cell damage was tested *in vitro* by culturing cells under hyperosmotic stress or by the addition of menadione, a generator of reactive oxygen species (ROS). Dry eye *in vivo* was induced by the injection of scopolamine into rats. Then, 930 nM of RS9 was applied to both eyes for 2 weeks. Oxidative stress was measured by the accumulation of 8-hydroxy-2’-deoxyguanosine (8-OHdG). Corneal wound healing was measured by scoring for superficial punctate keratitis (SPK). Corneal epithelial cell densities were evaluated histologically. RS9 and RTA 402 induced the expression of *NQO1* and *GCLC* mRNAs in HCE-T cells. And both compounds suppressed hyperosmotic-ROS generation and menadione induced cellular damage. However RS9 had a stronger protective effect than RTA 402. Ocular instillation of RS9 also significantly upregulated the expression of *Nqo1* mRNA in the corneal epithelium. Accumulation of 8-OHdG, increase of SPK scores and decrement of basal cell density were observed in corneal epithelium from scopolamine-injected rats. These changes were significantly ameliorated by the topical administration of RS9. RS9 induced Nfe2l2 activation and Nfe2l2-targeted genes, reduced oxidation, and ameliorated symptoms of dry eye using *in vitro* and *in vivo* models. Thus, RS9 might be a potent candidate agent against dry eye disease.

## Introduction

Dry eye is defined as a multifactorial disease of the ocular surface characterized by a loss of homeostasis of the tear film. It is accompanied by ocular symptoms including tear film instability and hyperosmolarity, ocular surface inflammation and damage, and neurosensory abnormalities, which have etiological roles [1]. Recent research progress has demonstrated that oxidative stress is involved in the pathology of dry eye. For example, increased levels of oxidation products were reported in brush cytology specimens from dry eye and Sjogren syndrome patients [2, 3]. In several rat dry eye models, an accumulation of oxidation products in the corneal epithelium was also reported [4, 5]. Reactive oxygen species (ROS) overproduction and accumulation of oxidation products were reported in an *in vitro* hyperosmotic stress model [6, 7]. Furthermore, Edaravone, a radical scavenger, protected primary corneal epithelial cells against hyperosmotic stress [8].

Mammalian cells have defense systems against oxidative stress under pathological conditions such as dry eye and cerebral ischemia. A central regulator of the stress response is nuclear factor-erythroid 2-related factor 2 (NFE2L2), a transcription factor, and its regulator kelch-like ECH-associated protein 1 (KEAP1), an adaptor component of the CUL3 (CULLIN 3)-based ubiquitin E3 ligase complex. Under physiological conditions, the NFE2L2 protein is maintained at a relatively low level because of its constitutive polyubiquitination mediated by KEAP1, which targets NFE2L2 for proteasomal degradation. When exposed to reactive oxygen species (ROS) and electrophiles, NFE2L2 dissociates from KEAP1, which leads to the stabilization, nuclear translocation, and accumulation of NFE2L2, followed by the upregulation of antioxidant gene expression [9]. NFE2L2 upregulates the expressions of antioxidants, genes associated with the glutathione and thioredoxin pathways, NADPH regenerating enzymes, and xenobiotic detoxification enzymes [10]. Two target mRNAs, *NAD(P)H dehydrogenase [quinone] 1* (*NQO1*) and *glutamate—cysteine ligase catalytic subunit* (*GCLC*) are often measured as indicators of NFE2L2 activation. *NQO1* removes quinone from biological systems as a detoxification reaction and *GCLC* is the first rate-limiting enzyme of glutathione synthesis.

Several NFE2L2 activators such as sulforaphane, bardoxolone methyl (RTA402), omaveloxolone (RTA 408), and dimethyl fumarate (DMF) have been developed in the USA; especially, bardoxolone methyl are developed in phase III for diabetic nephropathy, which is stronger activity than other NFE2L2 activators [11]. Recently, the same class of the potent novel NFE2L2 activator, RS9 was discovered in the microbial transformation products of bardoxolone methyl, RTA 402 [12]. Microbial transformation is used to obtain novel compounds that would be challenging to synthesize chemically. The effects of RS9 have demonstrated in several disease models including blood-retinal barrier permeability in rabbits [11], contact dermatitis in mice [13], brain injury after ischemia reperfusion in mice [14], thinning of the outer nuclear layer in Rhodopsin Pro347Leu rabbits [15], light-induced photoreceptor cell damage [16], secondary brain injury after intracerebral hemorrhage [17], and pathological ocular angiogenesis and hyperpermeability [18]. However, the efficacy of NFE2L2 activator on dry eye remains to be examined. Because oxidative stress has an important role in the pathology of dry eye, the NFE2L2 activator, RS9 is expected to recover the symptoms of dry eye.

Thus, in this study, we assessed the efficacy of RS9 against dry eye using *in vitro* and *in vivo* models. The parent compound RTA 402 was also used as a comparative control in some experiments.

## Materials and methods

### Cell culture and treatment

An SV40-adeno vector transformed corneal epithelial cell line, HCE-T, was originally generated by Kaoru Araki-Sasaki MD, PhD [19] and was supplied by RIKEN Cell Bank (Tsukuba, Ibaraki, Japan). HCE-T cells were maintained in DMEM/F12 containing 40 μg/ml gentamicin, 10 ng/ml EGF, 5 μg/ml insulin, and 5% FBS (Thermo Fisher Scientific, Waltham, Massachusetts, USA) in 95% air/5% CO_2_ at 37°C. The medium was changed every 2–3 days. Cells were routinely passaged at 80% to 90% confluence and split at a ratio of 1:9 by digestion in TrypLE regents (Thermo Fisher Scientific). For the RT-qPCR, menadione assay, and hyperosmotic experiments, HCE-T cells were seeded at 7.4×10^4^ cells/2 ml in 6-well plates, 5×10^3^ cells/100 μl in 96-well plates, or 1.5×10^4^ cells/200 μl in an 8-well ibidi μ-slide (ibidi, Madison, Wisconsin, USA), respectively. To confirm RNA induction by RT-PCR, the cells were treated with 10 nM or 100 nM (non-toxic concentrations) of RTA402 or RS9 for 48 hours at 37°C. To examine the inhibitory effect of RTA402 or RS9 against oxidative stress, the cells were pre-treated with compounds for 24 hours, and then were further incubated for 30 hours with compounds in the presence of 15 μM menadione. LDH release was assessed after 30 hours of incubation. Menadione is processed to an unstable semiquinone radical by a variety of reductive enzymes, and then the unstable semiquinones can readily enter into a redox cycle when molecular oxygen is present, causing a reformation of the quinone, with the concomitant generation of ROS [20]. Concentration of menadione was determined by testing the induction level of cell death by varying the concentration of menadione at 2-fold dilution. For the *in vitro* hyperosmotic dry eye model, hyperosmotic media (450 mOsM) was made up by adding NaCl and treated to cells for 4 hours at 37°C. The cells were preincubated with RTA402 or RS9 for 24 hours at 37°C before menadione or hyperosmotic treatment.

### RT-qPCR analysis

Cells were lysed with TRIZOL (Thermo Fisher Scientific). RNA was extracted using an RNeasy Mini Kit (Qiagen, Hilden, Germany). cDNA was synthesized from 1 μg of RNA by Superscripts II (Thermo Fisher Scientific). Quantitative PCR was performed using the real-time PCR 7500 (Thermo Fisher Scientific). The assay ID of Taqman gene expression assays used to detect each gene are as follows: Human *18S* ribosomal RNA: Hs03928985_g1, Human *NQO1*: Hs01045993_g1, Human *GCLC*: Hs00155249_m1 (Thermo Fisher Scientific). For *in vivo* experiments, The assay ID of Taqman gene expression assays used to detect each gene are as follows: Rat *Gapdh*: Rn01775763_g1, Rat *Nqo1*: Rn00566528_m1, Rat *Gclc*: Rn00689046_m1.

### Western blotting

Whole cell lysates were subjected to electrophoresis in 4–12% NuPAGE gel (ThermoFisher Scientific). Western blots were performed with primary antibodies listed below; ACTB (Sigma-Aldrich, France, #A1978), NQO1 (Abcam, Cambridge, UK, #ab34173), GCLC (Abcam, #ab41463). Anti-Mouse IgG (whole molecule)-Alkaline Phosphatase (Simga-Aldrich, #A3562) was used as secondary antibody for ACTB. Anti-Rabbit IgG, HRP conjugated (Merck, #NA934VS) was used as a secondary antibody for NQO1 and GCLC. EC Prime Western Blotting System (Merck, Darmstadt, Germany) and AP Conjugate Substrate Kit (BioRad, Calfornia, US) were used for detection. Chemiluminescence images were recorded with an ImageQuant LAS 4000 CCD camera system (GE Healthcare).

### Cell death

Cell death was detected as lactate dehydrogenase activity in medium using an LDH Cytotoxicity Detection Kit (Takara, Shiga, Japan) [21].

### Dihydroethidium (DHE) staining

For ROS measurements under hyperosmotic conditions, cells were incubated with 5 μM of DHE (Thermo Fisher Scientific) in DMEM/F12 for 10 minutes at 37°C. After the removal of DHE, cells were treated with 450 mOsM of hyperosmotic media for 4 hours at 37°C. The cells were then observed by a fluorescence microscope, Olympus IX71 (Olympus, Shinjuku, Tokyo, Japan).

### Animals

All animal experiments were approved by the Animal Care and Use Committee of Senju Laboratory of Ocular Science and experimental animals were handled in accordance with the ARVO Statement for the Use of Animals in Ophthalmic and Vision Research and the NIH Guiding Principles in the Care and Use of Animals (DHEW Publication, NIH 80–23).

Sprague-Dawley rats ware purchased from SLC Inc. (Hamamatsu, Shizuoka, Japan) and housed in a 12 h:12 h light-dark cycle and allowed free access to food and water. RS9 (9.3–930 nM) was dissolved in 0.1% (w/v) sodium dihydrogenphosphate dihydrate and 0.9% (w/v) sodium chloride (Nacalai Tesque, Kyoto, Japan) solution. The final pH was adjusted to 7.0 with NaOH (Wako, Chuo, Osaka, Japan). RS9 solution was prepared just before its administration. For RNA induction experiments in rats using RS9, 10 μl of RS9 eye drops was administrated to rat eyes six times at intervals of 2 hours. Two hours after the final administration, rats were euthanatized with an overdose of pentobarbital and corneal epithelial cells were collected by razor shaving.

### Scopolamine-induced rat dry eye model

Scopolamine is a muscarinic cholinergic antagonist that decreases tear secretion by inhibiting the parasympathetic nervous system [1]. Rat scopolamine-induced dry eye model was induced according to a previously reported method [22]. Scopolamine (Sigma-Aldrich, France) was continuously and systemically exposed to the animals at 12.5 mg/day by osmotic pump (Durect Corporation, Cupertino, California, USA). After 3 days of scopolamine exposure, 10 μl of 930 nM RS9 was topically administrated six times per day at intervals of 2 hours to both eyes of rats for 14 days. For RNA induction experiments in scopolamine-treated rats, corneal epithelial cells were collected using the same protocol as for normal rats.

### Superficial punctate keratitis (SPK) scoring of the cornea

After 14 days of RS9 topical administration to scopolamine-treated rats, they were anesthetized by inhaled induction of 3% isoflurane. SPK in the corneal surface was stained by applying a 0.1% fluorescein solution. After 30 seconds, extra fluorescein solution was washed out by saline solution. Corneas of anaesthetized animals were observed by slit lamp examination. The severity of punctate staining was evaluated using a modification of the method of Murakami et al [23]. Briefly, the corneal score of fluorescein infiltration was determined at each 1/3 area of the cornea (upper, intermediate, and lower). The score was categorized from 0 to 3 with 0.5 grade steps depending on the observation: 0 = no fluorescence, 1 = fluorescence resembling sparse dots, 2 = dense dot-like pattern, and 3 = very dense dot-like fluorescence. After SPK scoring, cornea were subjected to immunostaining, histological examination and RT-PCR respectively.

### Immunostaining of 8-hydroxy-2’-deoxyguanosine in the cornea

Rats were euthanatized with an overdose of pentobarbital and their eyeballs were removed and fixed in Zamboni solution (Wako, Tokyo, Japan) for 24 hours. After the elimination of extra structures, the cornea was immersed in blocking buffer containing 2% skimmed milk and 0.5% Triton X-100. Anti-DNA/RNA damage [15A3] (Abcam, #ab62623) was used as the primary antibody. Goat anti-mouse IgG (H+L) highly cross-adsorbed secondary antibody, Alexa fluor plus 488 was used as the secondary antibody (Thermo Fisher Scientific). After immunostaining, the cornea was cut and flat mounted with Vectashield (Vector Laboratories Inc. Burlingame, California, USA). Confocal microscopy using LSM 710 (Zeiss, Oberkochen, Germany) was used for image observation.

### Histological examination of the corneal epithelium

Rats were euthanatized with an overdose of pentobarbital and their eyeballs fixed in Zamboni solution (Wako) for 24 hours. Corneal flat mounts stained with TOPRO-3 (Thermo Fisher Scientific) were analyzed by confocal microscopy. The number of nuclei in the basal cell layer of the corneal epithelium was counted in an area of 15,000 μm^2^.

### Statistical analyses

Data are expressed as the mean ± standard deviation of the mean (SD), unless otherwise noted. Statistical analyses of *in vitro* experiments were performed using Dunnett’s test. Statistical analyses of *in vivo* experiments were performed using Dunnett’s test and Student’s *t*-test. Each statistical method was described in each figure legend. Statistical significance was defined when *p<0.05 and **p<0.01. Student’s *t*-test was used for comparison of the mean between two groups. On the other hand, Dunnett’s test is the multiple comparison test method when there is a control group and two or more treatment groups. Dunnett’s test was used to compare the mean of control and treatment groups.

## Results

### RS9 increases the expressions of NFE2L2-target genes in HCE-T cells

**Fig 1:**
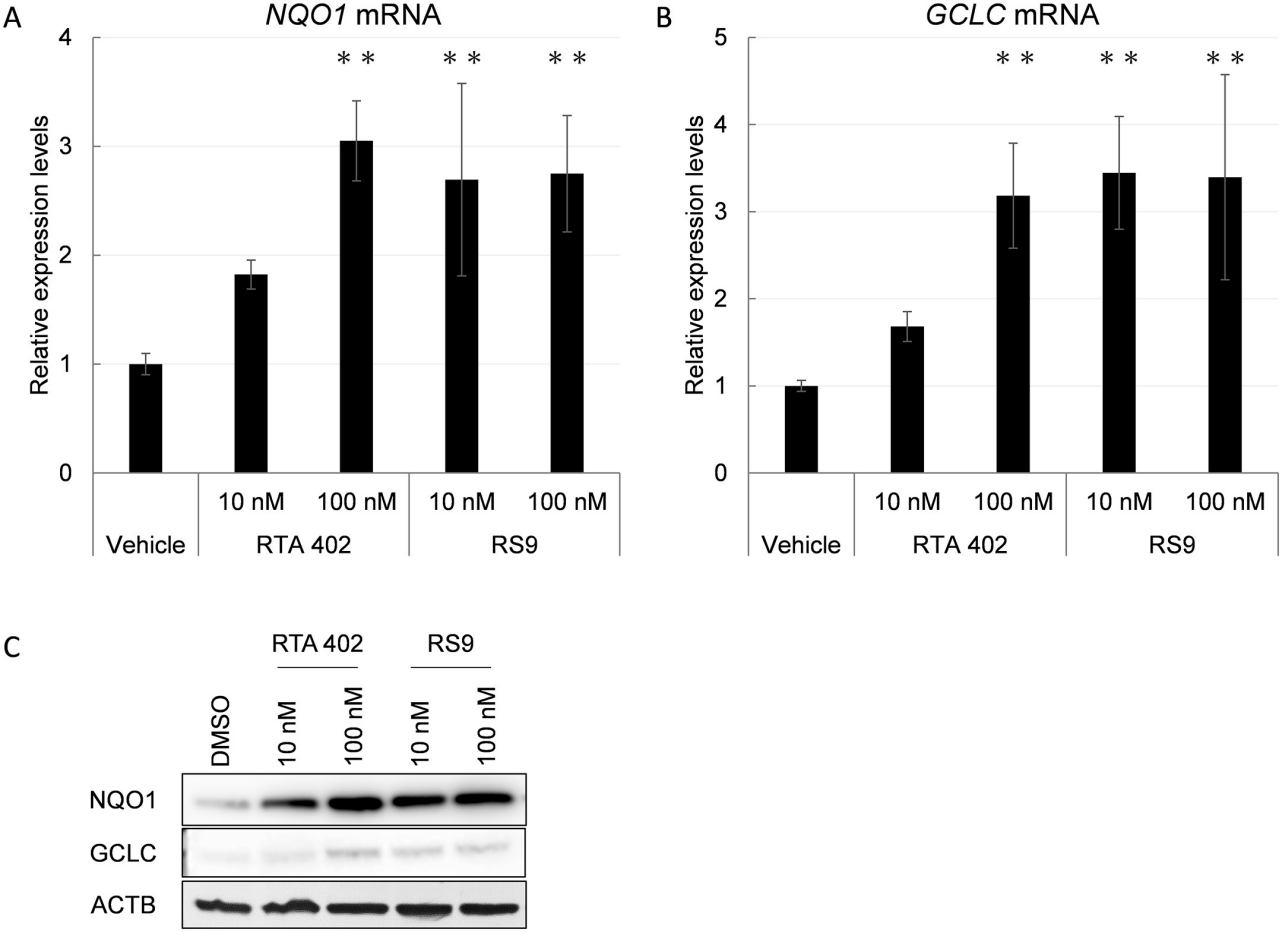
NFE2L2-targeted gene activation in HCE-T cells by RTA 402 and RS9. A, RT-qPCR analysis of *NAD(P)H dehydrogenase*, *quinone 1* (*NQO1*) mRNA and B, *Glutamate-cysteine ligase catalytic subunit* (*GCLC*) mRNA in HCE-T cells treated with RTA 402 and RS9. HCE-T cells were treated with 10 nM and 100 nM of RTA 402 and RS9 for 48 hours. Relative mRNA expression levels were calculated relative to vehicle-treated samples. RS9 induced *NQO1* and *GCLC* mRNA expressions at a lower concentration compared with RTA 402. Data are presented as the mean ± SD (N = 4). **P<0.01 compared with vehicle, Dunnett’s test. C, Western blot analysis of NQO1 and GCLC in HCE-T cells treated with RTA 402 and RS9. Whole cell lysates were collected after 48 hours treatment of RTA 402 or RS9. ACTB was detected as internal control. Western blot data confirmed NQO1 and GCLC inductions at protein levels. Representative data were shown (N = 2).

RS9 and its parent compound RTA 402, increased *NQO1* and *GCLC* mRNA expressions in HCE-T cells. As shown in Fig 1A & 1B, RS9 activated NFE2L2 and induced these gene expressions at a lower concentration than RTA 402 at 48 hours. We also confirmed induction of NQO1 and GCLC at protein levels by western blot (Fig 1C).

### RS9 inhibits menadione-induced cellular damage in HCE-T cells

**Fig 2:**
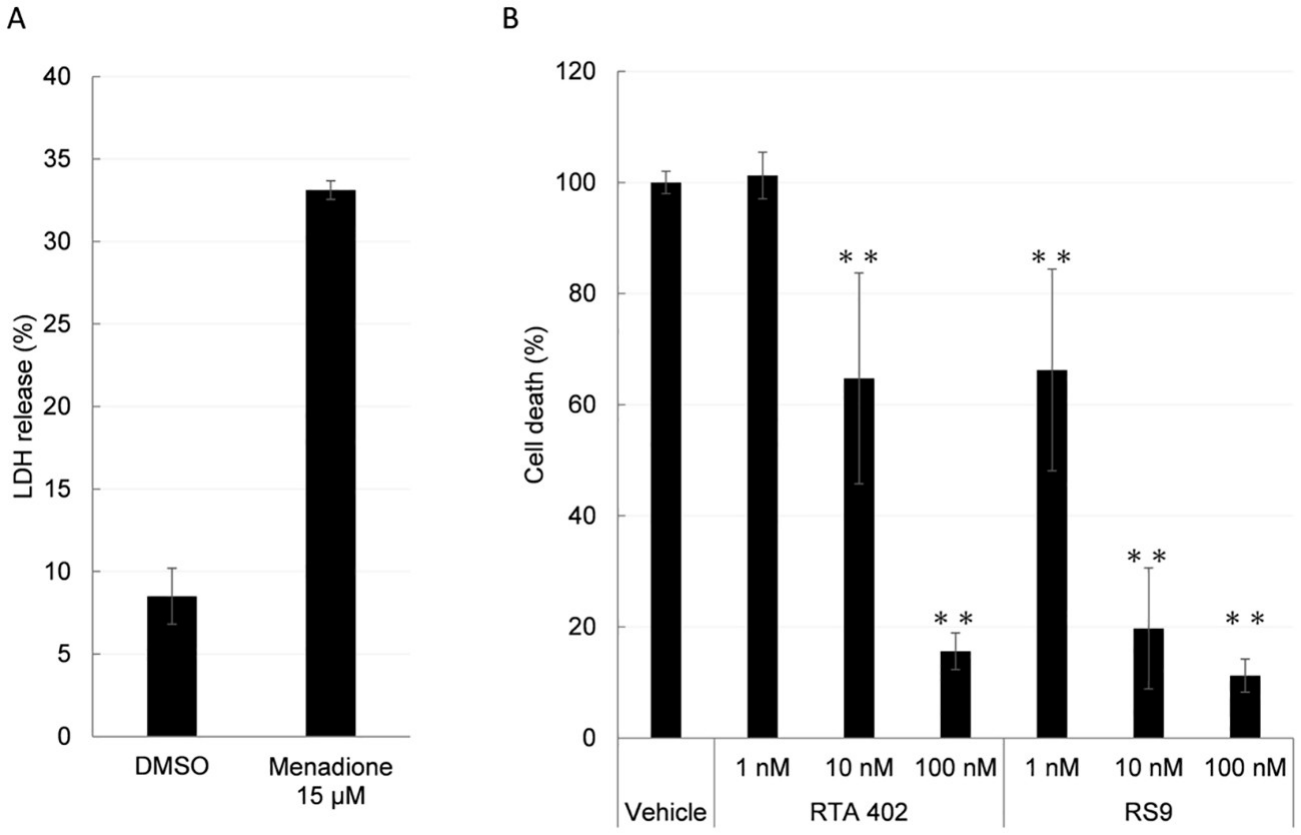
LDH cytotoxicity assay after menadione treatment. A, Cell death was induced by 15 μM of menadione. LDH release was measured 30 hours after menadione treatment. B, HCE-T cells were pre-treated with compounds for 24 hours, and then were further incubated for 30 hours with compounds in the presence of 15 μM menadione. LDH release was assessed after incubation. The percentage of cell death was calculated based on LDH release from normal and menadione-treated HCE-T cells. RTA 402 and RS9 pretreatment reduced cell damage in a concentration-dependent manner. RS9 protected against cell death at a lower concentration compared with RTA 402. Experiments were conducted in triplicate and repeated twice. Representative data are shown. Data are expressed as the means ± SD. **P<0.01 compared with vehicle under menadione treatment, Dunnett’s test.

To test protective effect of RS9 against oxidative stress, we used a ROS-inducer, menadione. After cells were treated with 15 μM of menadione for 30 hours, 30% LDH leakage was induced (Fig 2A). RTA 402 and RS9 protected against cellular damage in a concentration-dependent manner. RS9 exhibited protective potential at a lower concentration compared with RTA 402 (Fig 2B). The EC_50_ of RS9 and RTA 402 in this experiment was 1.7 nM and 15.5 nM, respectively. These data indicate RS9 is about 10-times more potent against oxidative stress than its parent compound.

### RS9 inhibits ROS overproduction in HCE-T cells exposed to hyperosmotic medium

**Fig 3:**
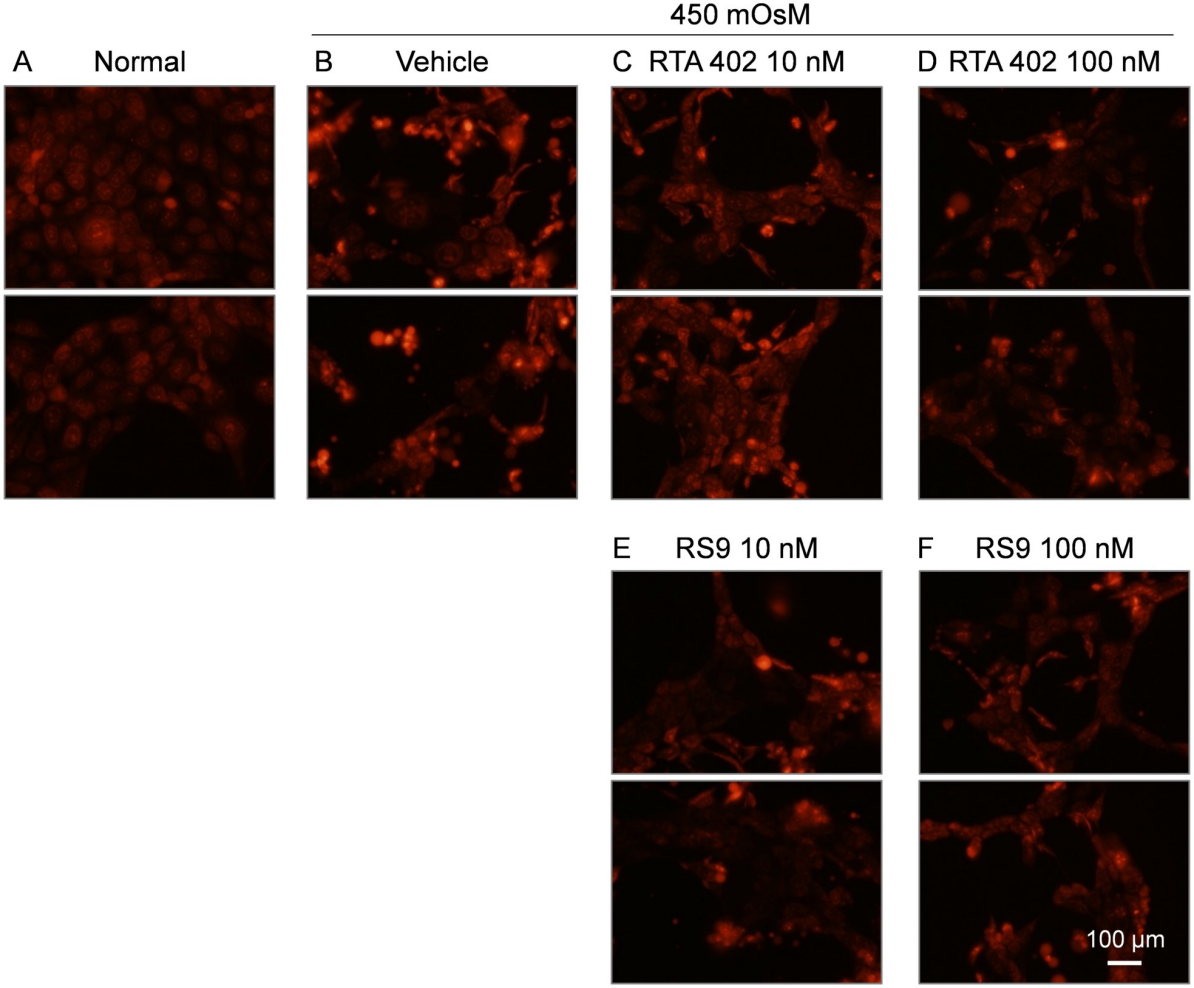
Inhibitory effects of RTA402 and RS9 on ROS production in HCE-T cells exposed to 450 mOsM of hyperosmotic medium. After 24 hours of RTA 402 or RS9 pretreatment, cells were exposed to 450 mOsM of hyperosmotic medium. Osmolarity was modified by addition of NaCl. ROS levels were detected by dihydroethidium (DHE) staining and analyzed by fluorescence microscopy 4 hours after the induction of hyperosmotic stress. Red color indicated oxidation form of DHE. A, Staining of cells under isosmotic culture conditions and B-F, staining under hyperosmotic culture conditions. ROS production was increased in cells exposed to hyperosmotic medium B, while ROS production was suppressed in RTA 402 or RS9 pretreated cells (C-F). Experiments were conducted in duplicate and repeated twice. Five images were obtained from one culture and representative images are shown.

Next, we tested whether RS9 inhibited ROS accumulation under hyperosmotic medium (450 mOsM) conditions as an *in vitro* dry eye model. Signal intensity of DHE staining for ROS measurement was increased at 4 hours after hyperosmotic stress (Fig 3A and 3B). Cells were shrunk due to osmotic stress in Fig 3B–3F. However, increased ROS production under hyperosmotic stress was inhibited in cells after pretreatment with 10 nM and 100 nM of RTA 402 or RS9 (Fig 3C–3F). These data indicate RTA 402 and RS9 inhibit ROS accumulation under hyperosmotic stress.

### Ocular topical instillation of RS9 increases Nfe2l2-targeted genes in the cornea

**Fig 4:**
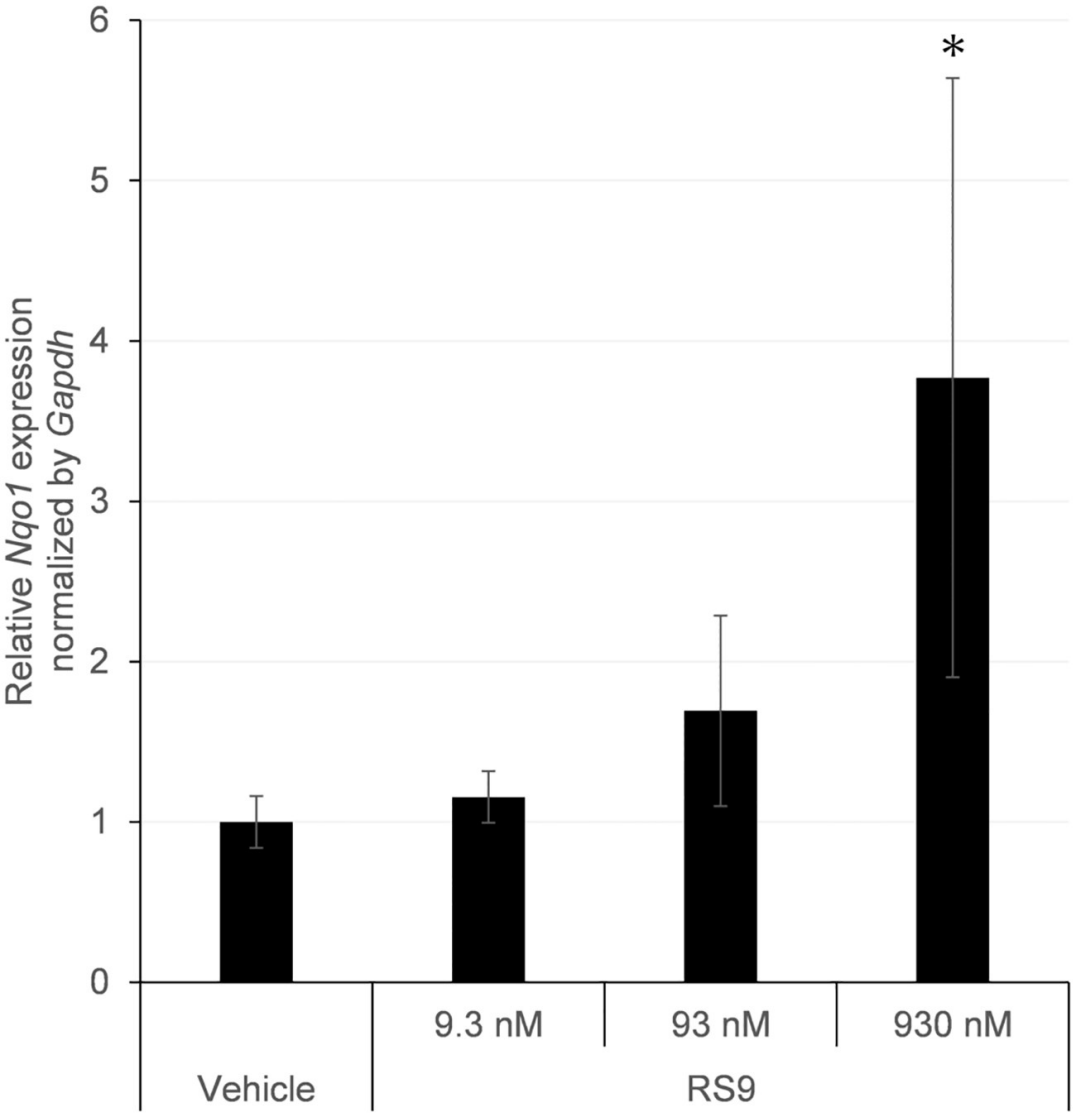
RT-qPCR analysis of *Nqo1* mRNA in corneal epithelium from rats treated with topical administration of RS9. RS9 was topically administrated to rat eyes at concentrations of 9.3–930 nM. Fold changes were compared with expression levels in the vehicle group. The topical administration of 930 nM RS9 significantly induced *Nqo1* mRNA expression. Data are presented as the mean ± SD (N = 6–7). *P<0.01, compared with vehicle, Dunnett’s test.

Since the quantity of protein in lysate from rat corneal epithelium is very small and the induction of proteins and mRNAs by compounds was well correlated *in vitro* (Fig 1). We conducted only mRNA experiments to detect *Nqo1* and *Gclc* in rat corneal epithelium. As shown in Fig 4, *Nqo1* mRNA levels were tend to increase in a concentration-dependent manner and significantly increased in the corneal epithelium after the instillation of 930 nM RS9 to normal rat eyes. In contrast, *Gclc* mRNA was not induced by RS9 in rat corneal epithelium (S1 Fig).

### RS9 prevents 8-OHdG accumulation and increases the SPK score in corneas of the scopolamine-induced dry eye model

**Fig 5:**
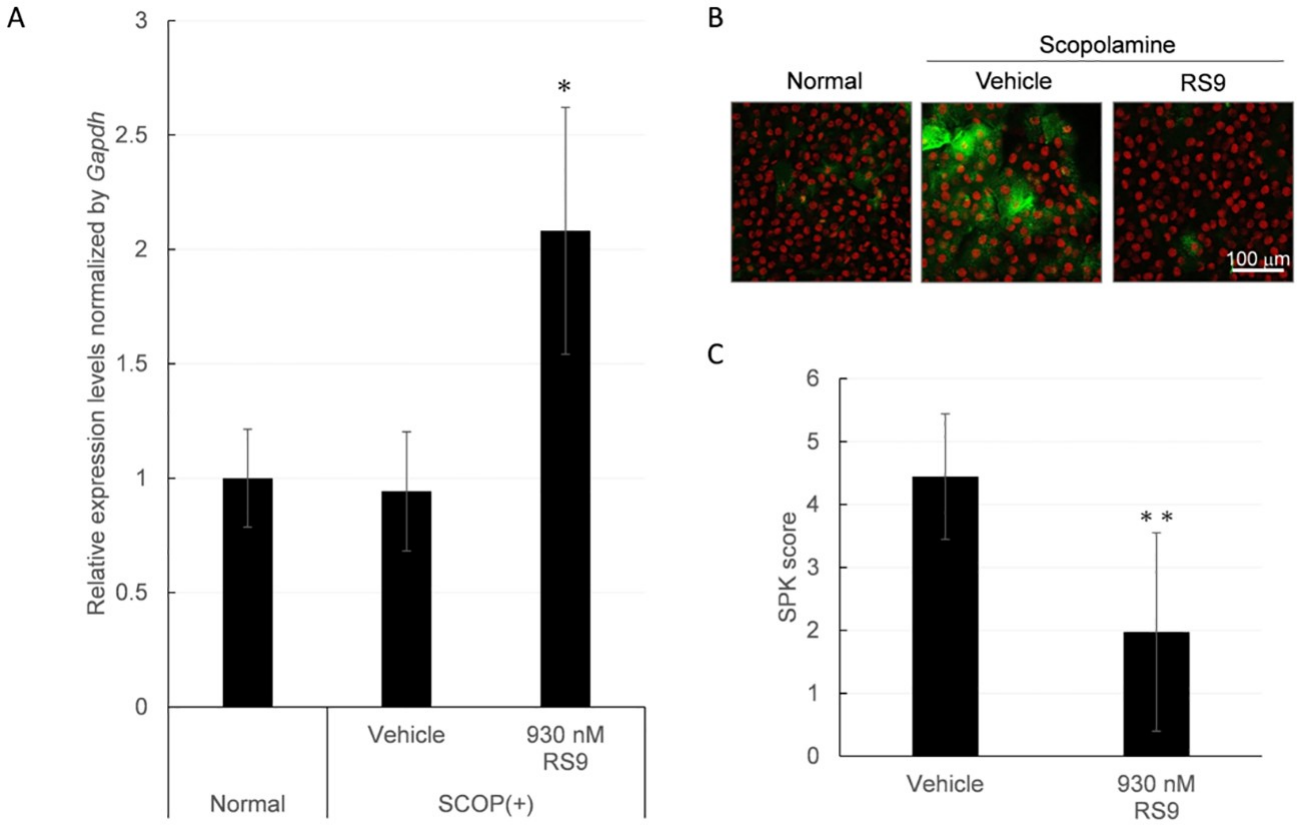
8-OHdG accumulation and increases the SPK score in corneas of the scopolamine-induced dry eye model after RS9 treatment. A, Induction of *Nqo1* mRNA and B, decrease in the oxidative stress marker, 8-OHdG and C, SPK score by RS9 in the corneal epithelium of scopolamine-treated rats. A, 2 weeks of topical administration of RS9 significantly induced *Nqo1* mRNA expression in scopolamine-treated rats. Data are presented as the mean ± SD (N = 2–6). *P<0.05, compared with normal. B, Immunohistochemistry of 8-OHdG was performed in the corneas of scopolamine-treated rats. Confocal microscopic analysis of corneal epithelial cells were performed after immunostaining of 8-OHdG (Green) and TOPRO nuclear staining (Red). Immunostaining of 8-OHdG accumulated in scopolamine-treated rats and was reduced by RS9 topical administration. Representative data are shown. C, Superficial punctate keratitis (SPK) score was evaluated by fluorescein staining. SPK scores were decreased after topical administration of RS9. Integrated data from two trials are shown. Data are the means ± SD (N = 10). **P<0.01, compared with vehicle, Student’s *t*-test.

We found that *Nqo1* mRNA expression levels were significantly upregulated in scopolamine rats receiving 930 nM of RS9 (Fig 5A). To confirm the oxidative stress in the corneal epithelium in this model, we assessed the increase of 8-hydroxy-2’-deoxyguanosine (8-OHdG). In scopolamine-treated rats, immunostaining of 8-OHdG was increased (Fig 5B, green signal in vehicle). When 930 nM of RS9 was topically administrated to scopolamine-treated rats, 8-OHdG positive staining was decreased (Fig 5B, RS9). As expected, RS9 topical administration significantly reduced SPK scores (Fig 5C, RS9). These data indicate mRNA induction by RS9 might correspond with the decrease in ROS production and SPK score in the *in vivo* dry eye model.

### RS9 recovers the density of corneal basal epithelial cells in scopolamine-treated rats

**Fig 6:**
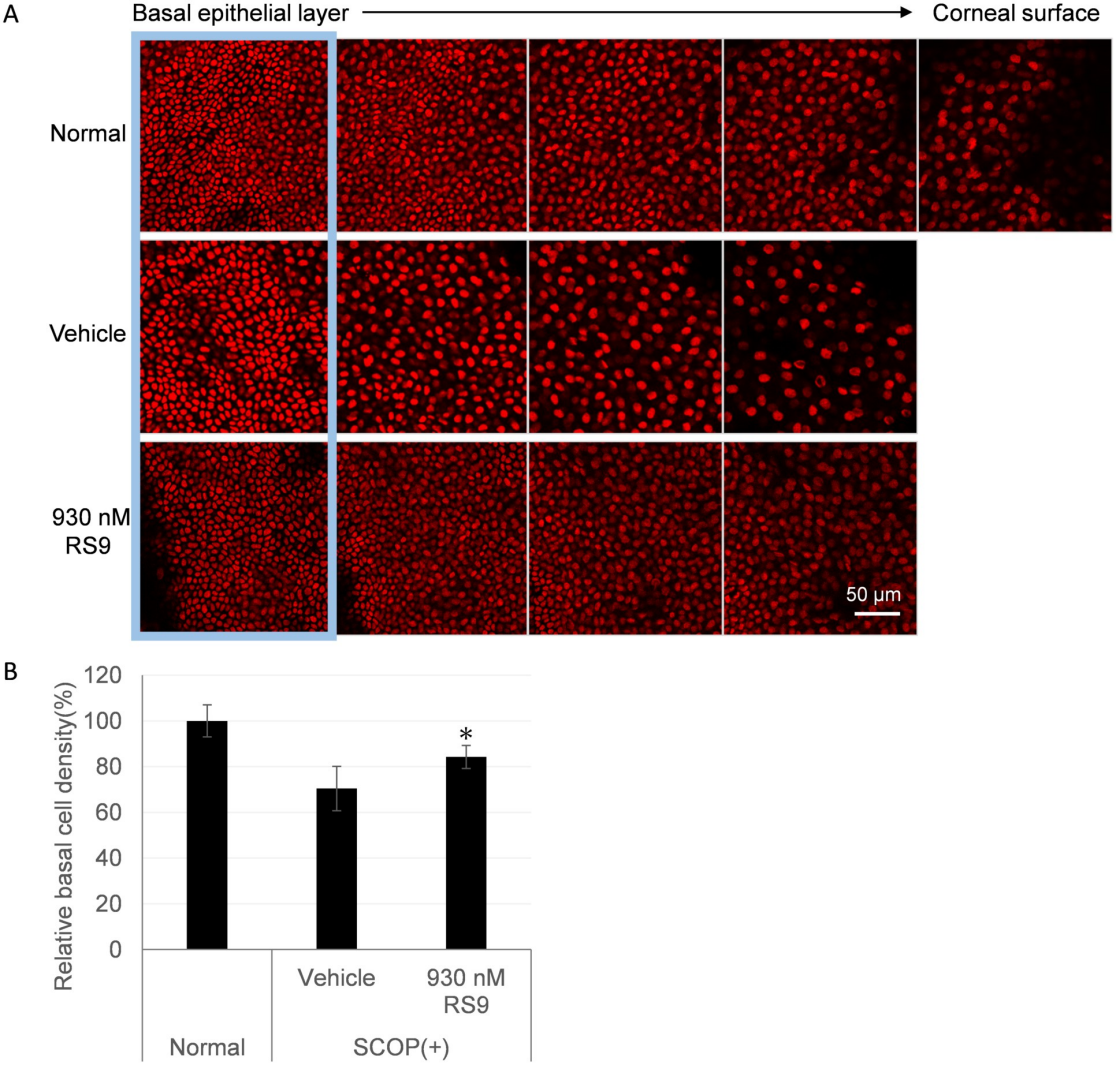
Cellular density of corneal epithelium in scopolamine-treated rats. A, Changes of density in corneal epithelial cells were analyzed by a corneal flat mount. Z-stack images every 5 μm from basal epithelial layer were shown (from left to right). Red color indicated nuclear stained with TOPRO. The blue square indicated the corneal basal epithelial cells layer. B, corneal basal epithelial cell density was evaluated in same area. Three areas were analyzed from one cornea. Density of basal epithelial cells were significantly recovered by RS9 topical administration (B). Data represent mean ± SD (n = 4~5); *P < 0.05, compared with vehicle, Student’s t test.

We investigated the effects of RS9 on histological changes of the cornea in scopolamine-treated rats. Cornea flat mount stained with TOPRO to observe nuclear. Every 5 μm Z-stack images of corneal epithelium were obtained to test density of nuclear in each layer. From the confocal microscopy observation, cells density of corneal epithelium was relatively decreased in scopolamine-treated rats and recovered by RS9 topical administration (Fig 6A). The number of basal corneal epithelial cells from scopolamine-treated rats had decreased to about 70% and that basal cells had larger nuclei (Fig 6B, vehicle). RS9 topical administration significantly recovered basal cell density to 80% (Fig 6B, RS9). These data suggest scopolamine treatment decreases not only superficial corneal epithelial cells observed by SPK but also the number of corneal basal epithelial cells, and that RS9 partially inhibits this decrease in cell number.

## Discussion

NFE2L2 is a key transcriptional factor that regulates the expression of anti-oxidant enzymes. In this study, we demonstrated that a novel potent NFE2L2 activator, RS9, induced NFE2L2-targetd genes: *NQO1* and *GCLC* mRNAs and protected cells against oxidation and cell death *in vitro*. Furthermore, we demonstrated that the topical administration of RS9 induced *Nqo1* mRNA expression, but not *Gclc* mRNA, and ameliorated the vicious symptoms of ocular surface in a rat dry eye model.

Nakagami et al. [11] has already reported that structural differences between RTA 402 and RS9 are epoxidation of A-ring and hydroxylation of E-ring, and hydroxylation in the E-ring, but not the epoxidation of A-ring of RS9 was involved in an improvement in NFE2L2 activation ability.

First, we used a hyperosmotic stress model as an in vitro dry eye model. Tear film osmolality is thought to be involved in the pathogenesis of aqueous deficient dry eye (ADDE) and evaporative dry eye (EDE). Evaporation during the blink interval causes a measurable thinning of the tear film, and a consequent rise in tear film osmolality was predicted [1]. As previously reported, in vitro hyperosmotic stress induced ROS overproduction in HCE-T cells (Fig 3B). RS9 pretreatment reduced ROS overproduction under hyperosmotic stress (Fig 3C–3F). These data indicate RS9 pretreatment induces anti-oxidant enzymes via NFE2L2 activation and reduces oxidative stress under hyperosmotic stress. This is first report to demonstrate the protective effects of an NFE2L2 direct-activator against hyperosmotic stress.

To analyze the efficacy of RS9 on ocular surface damage *in vivo*, we used a scopolamine-induced rat dry eye model. Inhibiting tear secretion by systemic administration of the muscarinic cholinergic antagonist scopolamine produced keratoconjunctivitis sicca that mimics human dry eye. This scopolamine-induced dry eye model is commonly used animal model in DED research [22, 24–27]. We demonstrated that the oxidation product 8-OHdG accumulated in the corneal epithelial cells of this model. This is a common phenomenon similar to other rat dry eye models such as the blink-suppressed model and lacrimal gland removal model [4, 5]. The topical administration of RS9 decreased 8-OHdG positive staining (Fig 5B). In addition, RS9 prevented the increase of SPK in the scopolamine-induced dry eye model (Fig 5C). According to previous reports [6, 7, 28], there might be a relationship between tear depleted-hyperosmolality, oxidative stress and SPK. However, oxidative stress might be also caused by the mechanical stress of blinking or by the direct effects of scopolamine. Further *in vivo* investigation of the correlations between tear hyperosmolality, oxidative stress and corneal damage are required.

We also demonstrated that the density of corneal epithelial basal cells was decreased in this animal model and that RS9 partially recovered this histological change (Fig 6). NFE2L2 upregulates the expression of enzymes related to metabolic and anti-apoptotic pathways as well as the anti-oxidative pathway. Thus, RS9 might maintain the viability and/or proliferation of corneal basal cells by stimulating the metabolic and anti-apoptotic pathways. Previous studies have reported a decrease in epithelial cell density in patients with dry eye, corneal allodynia, and herpes simplex virus keratitis [29, 30, 31]. These findings suggest this animal model might partly mimic human dry eye patients.

In summary, our results indicate that RS9 induces NFE2L2-targeted genes, reduces oxidation, and ameliorates the symptoms of dry eye in *in vitro* and *in vivo* models. Thus, RS9 may be a potent candidate agent against dry eye disease.

## Supporting information

S1 Fig RT-qPCR analysis of *Gclc* mRNA in corneal epithelium from rats treated with topical administration of RS9. RS9 was topically administrated to rat eyes at concentrations of 9.3–930 nM. *Gclc* mRNA levels were analyzed by Taqman gene expression assay. Fold changes were compared with expression levels in the vehicle group. The topical administration of RS9 didn’t increase *Gclc* mRNA expression levels. In rats treated by 930 nM of RS9, *Gclc* mRNA levels seemed to decrease. But there was no significant differences. Data are presented as the mean ± SD (N = 4).
(TIF)
