# Supplementary figures and images for: NFE2L2 activator RS9 protects against corneal epithelial cell damage in dry eye models
Source: PLoS One. 2020 Apr 22;15(4):e0229421. doi: 10.1371/journal.pone.0229421 (PMC7176120; doi:10.1371/journal.pone.0229421)

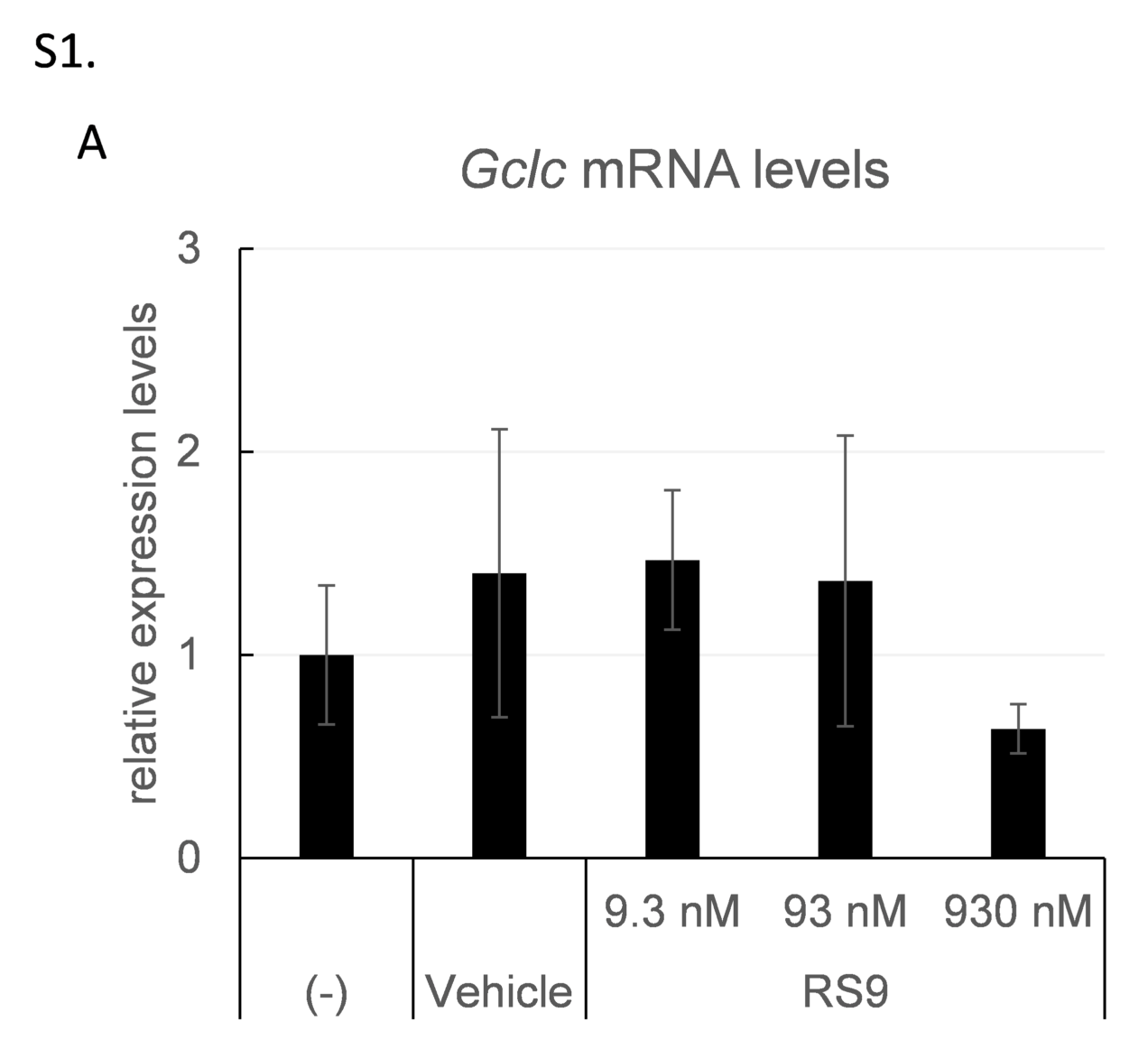

Supplement: S1 Fig — RS9 was topically administrated to rat eyes at concentrations of 9.3–930 nM. Gclc mRNA levels were analyzed by Taqman gene expression assay. Fold changes were compared with expression levels in the vehicle group. The topical administration of RS9 didn’t increase Gclc mRNA expression levels. In rats treated by 930 nM of RS9, Gclc mRNA levels seemed to decrease. But there was no significant differences. Data are presented as the mean ± SD (N = 4). (TIF) [file pone.0229421.s001.tif]
